# Study of the association of adrenomedullin and basic-fibroblast growth factors with the peripheral arterial blood flow and endothelial dysfunction biomarkers in type 2 diabetic patients with peripheral vascular insufficiency

**DOI:** 10.1186/s12929-014-0094-y

**Published:** 2014-10-07

**Authors:** Fawziah A Alrouq, Abeer A Al-Masri, Laila M AL-Dokhi, Khalid A Alregaiey, Nervana M Bayoumy, Faten A Zakareia

**Affiliations:** Physiology Department, Faculty of Medicine, King Saud University, Riyadh, Saudi Arabia

**Keywords:** Diabetic vasculopathy, Adrenomedullin, Basic-Fibroblast growth factor

## Abstract

**Background:**

Progressive micro-vascular vaso-degeneration is the major factor in progression of diabetic complications. Adrenomedullin (AM) and basic-Fibroblast growth factor (b-FGF) are strongly correlated with angiogenesis in vascular diseases. This study aims to provide base line data regarding the vascular effects and correlation of AM, and b-FGF with the peripheral blood flow in diabetic patients with peripheral vascular disease (PVD), and their effect on endothelial dysfunction markers. Ninety age- and sex matched females were enrolled in the study: 30 were controls, 30 had diabetes without complications (group II) and 30 had diabetes with PVD (group III) diagnosed by ankle/ brachial index (A/BI). Plasma levels of AM, b-FGF, intercellular adhesion molecule −1 (ICAM-1) and vascular cell adhesion molecule-1 (VCAM-1) were measured by indirect enzyme immunoassay (ELISA).

**Results:**

There was a significant increase in plasma AM, VCAM-1and ICAM-1, while a significant decrease in plasma b-FGF in diabetic patients with PVD (p < 0.05). A positive correlation was observed between plasma AM, b-FGF and A/BI and a negative correlation with VCAM −1 and ICAM in diabetic PVD. AM was not a predictor, while b-FG, VCAM-1 and ICAM-1 could be predictors for peripheral blood flow in diabetic PVD.

**Conclusion:**

This study elucidates for the first time that AM and b-FGF are correlated and have a direct impact on the peripheral blood flow, the rise of AM in diabetic PVD may be a consecutive and compensatory vasculo-protective effect as its angiogenic and anti-inflammatory properties act to relief the endothelial insult. Down expression of b-FGF may be a predisposing factor for micro-vascular derangement. It is not clear if the rise of AM and the decline of b- FGF levels may be consequences or predisposing factors for VCAM-1 and ICAM-1 elevation as these endothelial dysfunction biomarkers could reduce peripheral blood flow and vascular integrity. It is optimistic to believe that drug intervention through AM and b-FGF administration together with reversing the endothelial inflammatory process by targeting VCAM and ICAM could reduce the prevalence of diabetic vascular complications, reduce the risk of cerebrovascular and cardiovascular morbidity in diabetes through normalizing vascular endothelium function and peripheral blood flow.

## Background

Previous studies have confirmed the presence of micro -angiopathy characterized by basement membrane thickening, endothelial cell hyperplasia, hypertrophy, and pericyte cell degeneration in the diabetic state. Disruption of micro vascular blood flow may be among the earliest manifestations of diabetic neuropathy that ultimately contribute to the development of limb ulcers [1,2]. Hyperglycemia may potentiate the process of macro vascular lesion formation by inhibiting VSMC apoptosis, as well as increased cell proliferation that lead to a reduction in blood flow [3,4].

Adrenomedullin (AM) is a potent, long-lasting vasoactive, hypotensive peptide originally isolated from human pheochromocytoma .Smooth muscle and endothelial cells of the vasculature are major sites of AM synthesis and release [5]. AM is involved in a wide range of physiological processes, including vasodilatation, angiogenesis, inhibition of apoptosis and cell growth regulation .AM has an anti proliferative effects, it is an associated factor in the course of vascular and proliferative retinal diseases . AM protects a variety of cells against oxidative stress induced by stressors [6], it suppress oxidative stress through c-AMP signalling pathway [7,8]. Adrenomedullin (AM) is an endogenous peptide first identified as a strong vasodilating molecule. In mice, homozygous knockout of AM (*AM*−/−) or its receptor regulating protein, RAMP2 (*RAMP2*−/−), is embryonically lethal due to abnormal vascular development .AM expression in the retina is strongly induced by ischemia. However, AM enhanced the proliferation and migration of retinal endothelial cells [9]. Finally, it was found that injection of anti-AM antibody in vitrous humor reduced pathological retinal angiogenesis. It was concluded that AM and its receptor system is crucially involved in retinal angiogenesis and they are potential therapeutic targets for controlling pathological retinal angiogenesis [9].

There is lack of knowledge about AM precise role, regulation, production and release at the systemic level, and its correlation with the peripheral blood flow in diabetic vascular insult.

Fibroblast growth factor (b-FGF) has been widely reported to increase blood flow and promote angiogenesis in myocardium and peripheral vessels in animal models of vascular insufficiency [10].It stimulates angiogenesis, is a vasodilator , has anti apoptotic effects , and induces proliferation in various kinds of cells . b- FGF has been investigated in the field of wound healing, bone regeneration, acute ischemic models, and myocardial infarction. The angiogenic protein basic fibroblast growth factor (b-FGF; FGF-2) is able to enhance the collateral-dependent blood flow after bilateral femoral artery ligation in rats. Normal NO production is essential for the enhanced vascular remodeling induced by exogenous b-FGF in rat model of experimental peripheral arterial insufficiency. These results imply that a blunted endothelial NO production could temper vascular remodeling in response to b-FGF as an angiogenic growth factor [11].

Furthermore, its effects have been reported in the central and peripheral nervous system [12], however, its role in diabetic vasculopathy has not been studied before and is unclear.

ICAM-1 and VCAM-1 are important markers of endothelial dysfunction that have been demonstrated to play important roles in the development of diabetic retinopathy [13]. ICAM-1 and VCAM-1 mediate leukocyte adhesion to the retinal vasculature, that induces capillary occlusion which has a critical role in the development of diabetic retinopathy (DR) [14–16]. The expression of adhesion molecules can be induced by some pro-inflammatory cytokines like tumor necrosis factor- α (TNF-α) [17]. There is lack of evidences concerning the potential role of these two vascular adhesion molecules on the peripheral vascular blood flow in diabetic PVD.

Ankle/brachial index (A/BI) is a known noninvasive approach used for assessing peripheral arterial blood flow. Patients with an A/BI of 0.7–0.9 and <0.7 have high hazard ratios for all cause mortality [18]. Abnormal A/BI is associated with an increase in prevalence of stroke .A low ABI of ≤0.9 is more likely in patients with a history of PVD [19].

Despite the extensive animal work on the role of endothelial dysfunction in diabetic complications, data pertaining to any possible association between diabetic PVD and different angiogenic, anti-inflammatory markers in human studies are insufficient. Therefore, this study aims to correlate the circulating AM and b-FGF to the ankle/brachial index as a marker of the peripheral blood flow in patients with type 2 diabetes mellitus with PVD and with the levels of endothelial inflammatory markers.

## Methods

### Subjects

This study was carried out from March 2008 to November 2011 at the Clinical Physiology Laboratory and Diabetes Research Center of King Abdul Aziz University Hospital (Riyadh, Saudi Arabia). All biochemical parameters were measured in a biochemistry laboratory at the Physiology Department (King Khalid University Hospital). The study was approved by the Ethics Committee of King Khalid University Hospital, and all of the procedures were performed in accordance with ethical approval institutional guidelines. The study protocol followed the ethical guidelines of the most recent Declaration of Helsinki. Written consent was obtained from the participants prior to the start of the study.

The total number of age and sex matched patients with type 2 diabetes was 150, recruited from Diabetes Research Centre of King Abdul Aziz University Hospital, they were subjected to history taking, thorough clinical examination , and laboratory investigations to establish diagnosis of type 2 diabetes and PVD , also to exclude type 1 diabetes, other diabetic complications, and associated pathological conditions. 50 patients were excluded from the study as they have other diabetic complications as mentioned in the exclusion section, 15 had retinopathy, 20 had nephropathy and 15 with diabetic neuropathy as diagnosed by the laboratory investigations, fundus ophthalmoscopy and clinical neurological examination. Out of the remaining 100 patients we have been chosen 30 without any diabetic complications and 30 patients with diabetic PVD. Concerning the procedures for determining the diagnosis of PVD, it was assessed by consultants of clinical physiology by clinical examination and measurement of Lower-limb blood flow and A/BI. Micro vascular blood flow was accurately measured noninvasively using continuous Doppler flowmetry (MD6 System, Hokanson, WA, USA) for measurements of A/BI [16] as mentioned below.

Relatives of the diabetic patients were invited to participate in the study. They were recruited by social workers and nurses in the outpatient unit from Diabetes Research Centre of King Abdul Aziz University Hospital (Riyadh, Saudi Arabia), from March 2008 to November 2011, they were age and sex matched to patients groups, were not receiving any medication. Relatives received information about the study and its aims and gave their informed consent to participate. The control group consisted of relatives who had initially agreed to participate. The number of relatives wishing to participate was too low for a random selection. There was high rates of refusal and dropout from groups for diabetic patients relatives. Although not chosen at random, all participants in the control group had initially wished to join the control group, so, presumably, they constituted a satisfactory control group. Of the 150 eligible relatives approached, 50 refused to participate in this study, 25 were excluded due to the presence of hypertension and cardiovascular problems, 75 relatives were assigned to the control group participants. Out of them 45 declined to continue participating after the group started and the remaining 30 subjects were included in the study.

The present study was carried out on 90 adult females, of the same age group, divided into three groups according to the following experimental design:

- Group I: 30 healthy control females
- Group II: 30 Type 2 controlled diabetes patients (without complications such as retinopathy, nephropathy or neuropathy)
- Group III: 30 Type 2 diabetic patients with PVD, as diagnosed by A/BI

### Diabetic patients fulfilled the following inclusion criteria

They were all Type 2 diabetic patients. Metabolic control was assessed on the basis of Glycosylated HbA1c level. They were on oral hypoglycemics. They had normal blood pressure, kidney function and lipid profile. All patients were examined for the presence of diabetic PVD by A/BI.

Subjects with the following exclusion disorders were excluded from the study.

- Patients with Type 1 diabetes, diagnosed by history and clinical examination
- Diabetic nephropathy or renal failure .Urinary albumin excretion 30–300 mg/24 h Was regarded as microalbuminuria.
- Patients with diabetic retinopathy, diagnosed by fundus ophthalmoscopy
- Hypothyroidism or hyperthyroidism, diagnosed by abnormal thyroid function tests
- Presence of coronary artery disease or congestive heart failure, diagnosed by ECG abnormalities
- Pregnancy and presence of any psychological or neurological disorder.

### Sample collection

All control and diabetic individuals fasted for 8 h and 10 ml of their blood was collected in EDTA tubes, centrifuged at 3500 rpm for10 min for plasma collection and stored at −70°C. Plasma samples were analyzed for AM, b-FGF, VCAM-1, and ICAM-1. Samples were assayed in duplicate in a single large batch. Measurement kits were purchased from R & D Systems (Minneapolis, MN, USA).

### Parameters measured

#### Routine investigations

Measurements of fasting and postprandial serum glucose level [20], and glycosylated hemoglobin (HbA1C).Whole blood was used for the assessment and was analyzed by high-pressure liquid chromatography [21]. Kidney function tests, complete urine analysis and detection of microalbuminuria [20] were carried out.

### Specific investigations

#### AM by indirect enzyme immunoassay (ELISA)

All reagents and standards were prepared as instructed in the kit. A 100-μl assay diluents RD1W (a buffered protein solution with preservatives), along with 100 μl of standard, control or sample were added to each well and incubated for 2 h at room temperature. The wells were aspirated and washed three times. A total of 200 μl of AM conjugate was added to each well, covered and incubated for 2 h at room temperature, then aspirated and washed three times. Substrate solution (200 μl) was then added to each well, which were protected from light and incubated for 25 min at room temperature. A total of 50 μl of stop solution was then added to each well. This product was read at 450 nm using a micro plate reader within 30 min. The intensity of the produced colored product was directly proportional to the concentration of AM present in the samples. The results were calculated as directed in the kit [22].

### b-FGF, VCAM-1 and ICAM-1 by indirect enzyme immunoassay (ELISA)

All reagents and standards were prepared as instructed in the kit. A 100-μl assay diluents RD1W (a buffered protein solution with preservatives), along with 100 μl of standard, control or sample were added to each well and incubated for 2 h at room temperature. The wells were aspirated and washed three times. A total of 200 μl of the **b-FGF, or VCAM-1 or ICAM-1** conjugate was added to each well, covered and incubated for 2 h at room temperature, then aspirated and washed three times. Substrate solution (200 μl) was then added to each well, which were protected from light and incubated for 25 min at room temperature. A total of 50 μl of stop solution was then added to each well. This product was read at 450 nm using a micro plate reader within 30 min. The intensity of the produced colored product was directly proportional to the concentration of **b-FGF, VCAM-1 and ICAM-1** present in the samples. The results were calculated as directed in the kit [23].

### Lower-limb blood flow (A/BI)

Micro vascular blood flow was accurately measured noninvasively using continuous Doppler flowmetry (MD6 System, Hokanson, WA, USA) for measurements of A/BI [16]. Both brachial and ankle pressures were measured and used to calculate the A/BI. A/BI between 0.9 and 1.2 considered normal (free from significant PVD). PVD is defined as ≤ 0.9 which is 95% sensitive and 90% specific to for the presence of a ≥ 50% narrowing of a lower extremity artery, and is used to establish a diagnosis of PVD [16].

### Statistical analysis

All data are reported as mean ± standard deviation. Two-way analysis of variance was performed to compare the study groups, with differences considered significant for p < 0.05, with 95% Confidence Intervals. The Spearman rho correlation coefficient was applied to find the strength of correlation between continuous quantitative variables. Linear regression analysis was performed for AM, b-FGF, VCAM and ICAM (as independent variables), and A/BI (as a dependent variable). All statistical analysis performed with SAS 9.2 statistical software (version SAS 9.2 Institute, Inc, Cary, North Carolina, USA).

## Results

### Demographic features of the study

There was no significant change in disease duration in diabetic patients group II and III (9.7 ± 0.1 and 10.3 ± 1.0 years, respectively) (p = 0.06). On the screening day, diabetic patients group III had a significant increase in fasting blood glucose, which was 9.6 ± 1.7 mmol/dl compared to group II (7.1 ± 1.65) and III (p = 0.001). Also, a significant increase was observed in the postprandial serum glucose level, which was 10.4 ± 0.62 in group III and 7.1 ± 0.41 mmol/dl in group II (p = 0.001, p = 0.003). Also, there was a significant increase in HbA1c% which was 9.4 ± 0.2 in group III and 6.6 ± 0.1% in group II respectively) (p = 0.001, p = 0.002). Other screening measures were normal in groups II and III, including lipid profile (total cholesterol 5.2 ± 0.2 and 5.5 ± 1.1 mmol/l and triglycerides 1.9 ± 0.04 and 2.08 ± 0.04 mmol/l, respectively. Control subjects on the screening day had normal data as in Table 1.

**Table 1:** **Demographic and biochemical features of the study groups**

**Demographic and biochemical features**	**Group I controls** **(n** = **30)**	**Group II diabetics without complications** **(n** = **30)**	**Group III diabetics with PVD** **(n** = **30)**
Mean age (years)	51 ± 0.1	50 ± 0.9	51 ± 1.1
Fasting blood glucose level (mmol/dl)	5.7 ± 0.1	6.7 ± 1.65	9.6 ± 1.7*^¥^
Postprandial serum glucose level (mmol/dl)	5.8 ± 0.11	7.1 ± 0.41	10.4 ± 0.62*^¥^
Disease duration (years)		9.7 ± 0.1	10.3 ± 1.0
HbA1c (%)	5.5 ± 0.2	6.6 ± 0.2	9.4 ± 0.2*
*Lipid profile*			
Cholesterol (mmol/l)	4.8 ± 0.3	5.2 ± 0.2	5.5 ± 1.1
Triglycerides (mmol/l) 2.01 ± 0.09	1.9 ± 0.3	1.9 ± 0.04	2.08 ± 0.04
BMI (kg/m2)	25 ± 1.4	29 ± 1.5*	30 ± 1.1*
Kidney function			
Creatinine (μmol/l)	87.9 ± 4.6	93.1 ± 2.14	99.7 ± 1.8
Urea (mmol/l)	4.5 ± 0.6	5.0 ± 0.13	5.7 ± 0.6
Treatment		Oral hypoglycemics (metformin and/or gliclazide)	Oral hypoglycemics (metformin and/or gliclazide)

Results are expressed as mean ± standard deviation.

*Significant changes compared with control (p < 0.05).

^¥^Significant changes in comparison to diabetic Group II (P < .0.05).

### Ankle/brachial index (A/BI)

A/BI showed a significant decrease in group III diabetic patients with PVD compared with groups I and II. It was 0.73 ± 0.06 vs 1.03 ± 0.08 and 1.06 ± 0.06 respectively (p < 0.05 and p < 0.001, respectively) (Table 2).

**Table 2:** **Ankle**/**brachial index and**, **plasma levels of ADM**, **b**-**FGF and intercellular adhesion molecules**, **in all study groups**

**Parameter**	**Control N** **=** **30**	**Diabetics without complications N** **=** **30**	**Diabetics with Vasculopathy N** **=** **30**	**P value**
-Plasma ADM (pmol/ml)	2.96 ± 0.62	2.56 ± 0.5	5.66 ± 1.31*^¥^	P = 0.001*
				P = 0.01¥
-Plasma b- FGF (pg/ml)	3.96 ± 0.6	3.56 ± 0.5	1.66 ± 0.14	P = 0.002*
				P = 0.01¥
-Plasma ICAM-1 (pg/ml)	318.66 ± 35.19	349.87 ± 27.18	550.3 ± 15.4*^¥^	P = 0.004*
				P = 0.001¥
-Plasma VCAM-1(pg/ml)	677.85 ± 17.63	690.64 ± 15.081	863 ± 32.37*^¥^	P = 0.001*
				P = 0.001¥
-Ankle/brachial index	1.03 ± 0.08	1.06 ± 0.06	0.73 ± 0.06*^¥^	P = 0.003*
				P = 0.001¥

Results are expressed as mean ± S.D.

*Significant changes in comparison to control ( P < 0.05).

^¥^Significant changes in comparison to diabetic Group II (P < 0.05).

### Plasma levels of plasma AM, b-FGF, VCAM-1, and ICAM-1

**Figure 1:**
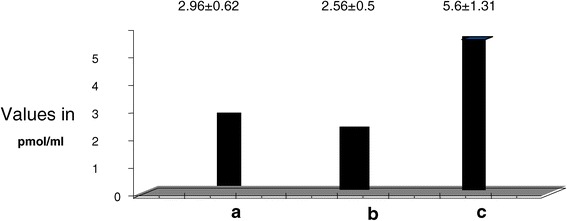
**Plasma Adrenomedullin in all study groups.** a. Control b. Diabetics without complications c. Diabetics with vasculopathy.

**Figure 2:**
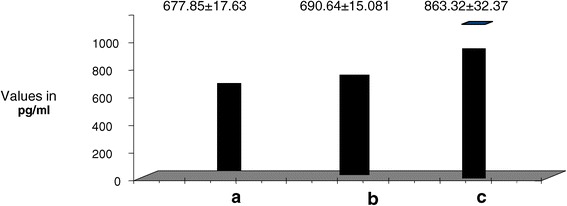
**Plasma VCAM-1 in all study groups.** a. Control b. Diabetics without complications c. Diabetics with vasculopathy.

**Figure 3:**
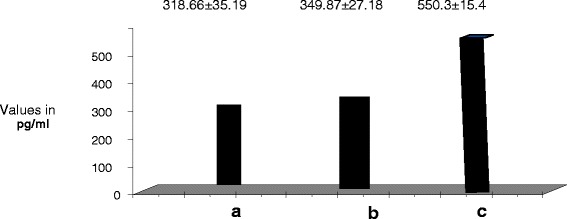
**Plasma ICAM in all study groups.** a. Control b. Diabetics without complications c. Diabetics with vasculopathy.

There was significant increase of plasma AM, VCAM-1, and ICAM-1 in diabetic patients with PVD compared to both control and diabetics without complications. AM was 5.66 ± 1.31 in group III vs 2.96 ± 0.6 and 3.56 ± 0.5 in groups I and II respectively. (p < 0.05). Concerning plasma ICAM-1 it was 550.3 ± 15.4 in group III vs 318.66 ± 35.19 and 349.87 ± 27.18 in groups I and II respectively (p < 0.05). Concerning plasma VCAM-1, it was 863.59 ± 32.37 in group III vs 677.85 ± 17.63 and 690.64 ± 15.081 in groups I and II respectively. (p < 0.05). Concerning Plasma b-FGF levels they were significantly decreased in diabetic patients with PVD compared to both control and diabetics without complications. b-FGF was 1.66 ± 0.14 in group III vs 3.96 ± 0.6 and 3.56 ± 0.5 in groups I and II respectively (p < 0.05). (Table 2, Figures 1, 2, 3).

### Correlations and linear regression

In group III, there was a positive correlation between A/BI and plasma AM and b-FGF (r =0.633) (p <0.001*), \(r =0.512) (p <0.001*) respectively, but there was a negative correlation with plasma VCAM-1 (r = −0.688, p < 0.013*), and ICAM (r = −0.787, p < 0.019*). Linear regression analysis was done for AM, b-FGF, VCAM-1, and ICAM (as independent variables), and A/BI as (dependent variables) in group III. AM was not a predictor for A/BI at least in this study, beta coefficient = 0.243 (p = 0.3). On the other hand, the analysis revealed b-FGF, VCAM-1 and ICAM-1 as predictors for A/BI in PVD, b-FGF beta coefficient = 0.577 (p = 0.036*), VCAM-1 beta coefficient = − 0.643 (p = 0.012*), for ICAM-1 beta coefficient = − 0.598 (p = 0.034*) (Table 3).

**Table 3:** **The correlation between different variables in group III**

**Diabetic vasculopathy**	**Correlation coefficient®**	**P value**
1- A/BI-- plasma ADM	0.633	<0.001*
2- A/BI-- plasma b-FGF	0.512	<0.01*
3-A/BI-- plasma VCAM-1	−0.688	<0.013*
4-A/BI-- plasma ICAM-1	−0.787	<0.019*

*Significant correlation.

## Discussion

We have previously [23] reported that AM was high in diabetic patients with retinopathy which explained based on the fact that AM is a multifunctional protective vasorelaxant peptide, and it was suggested as a possible newly protective factor in the course of vascular and proliferative retinal diseases. In the current study we extended the work on AM to include diabetic patients with PVD reporting the rise of plasma AM concentrations and its positive correlation with peripheral blood flow. These findings could be explained on basis that AM rise is a consequence of PVD to play an important vasodilator and angiogenic role in compensating the effects of vasoconstrictive molecules to ameliorate the peripheral blood flow. Previous investigations have proven that in diabetic angiopathies the levels of vasoconstrictive factors and AM are increased [24]. The raised concentration of AM and its positive correlation with A/BI in diabetic PVD shown in this study could demonstrate a novel association between AM and the vascular function, it may act to vasodilate, to induce an angiogenic and an anti-proliferative effects on the peripheral blood vessels and hence increases the peripheral blood flow, [24] AM could improve the vascular integrity through its anti-inflammatory effect [6,8]. Therefore, strategies increasing AM levels may be beneficial in diabetic PVD.

Oxidative stress has been shown to stimulate secretion of AM from endothelium and vascular smooth muscle cells [25]. The rise of plasma AM levels in diabetic PVD may be compensatory protective effect to the vasculature to compensate for oxidative stress-induced vasoconstriction , as shown in the study of Shimosawa et al. [25] who reported that endogenous AM may protect from organ injury by inhibiting oxidative stress production.

There is further sufficient evidence that augmented serum levels of AM in diabetic patients with PVD possibly result in response to hyperglycemia. Hayashi et al. [26] reported that plasma AM levels in patients with poorly controlled diabetes are significantly higher than those in healthy volunteers and proposed that to be from vascular AM expression induced by hyperglycemia. This fact could be beneficial to explain the high AM levels in diabetic patients with PVD who have poor glycemic control in this study as indexed with high HbA1c%.

Sakurai et al. [27] reported that AM ameliorates ischemia reperfusion (I/R) injury in rat livers by the reduction of oxidative stress, inhibition of apoptosis and the systemic inflammatory response. So, the augmented rise of AM in the current study supports the anti-oxidative, and anti-apoptotic effects of AM.

The study of Talero et al. [28] concluded that AM has a potential anti-inflammatory actions in the induced pleurisy, it attenuates the expression of proinflammatory cytokines, together with the decrease in neutrophil migration and oxidative stress during the inflammatory response. Taken together, these results with the result of the present study which shows a rise of AM together with ICAM and VCAM the well known endothelial inflammatory markers enhance understanding of the compensatory role of AM in counteracting the diabetic endothelial inflammatory dysfunction, and its role to improve the peripheral micro circulation.

Several experimental evidences point to a role for various FGFs in the neovascularisation process that takes place in inflammation, and angioproliferative diseases [29]. The significant decrease of plasma basic-FGF levels and the positive correlation with the peripheral blood flow in diabetic PVD reported in this study may be predisposing to the vascular derangement, endothelial dysfunction and raised endothelial inflammatory markers and may be the causative factor to the shortage in peripheral blood flow. These findings is in agreement with the findings of Asai et al. [30] who stated that b-FGF increases blood flow and promotes angiogenesis in myocardium and peripheral vessels in animal models of vascular insufficiency. Treatment of diabetic gangrene with topical application of a mixture of peripheral blood mononuclear cells (PBMC) and b-FGF resulted in a dramatic improvement in a short time. They concluded that this mixture appears to be a useful and a non-invasive treatment of diabetic gangrene [30].

Members of the FGF family can induce in vitro a complex proangiogenic phenotype in endothelial cells including the modulation of endothelial cell proliferation [31]. Interfering with FGF signaling could possibly leads to inhibition of angiogenesis. FGF2 null mice have a delay in wound healing which may be explained by inhibition of endothelial cell migration [32]. FGFs are able to promote angiogenesis and the formation of collateral circulation in cardiac or hind limb models of ischemia, the administration of FGF2 protein increases the formation of new vessels and collaterals [32].

So, it is conceivable to explain that lack of FGF in this study predisposes for the shortage of the peripheral blood flow, due to lack of peripheral neoangiogenesis, formation of neovessels and collaterals as shown by low A/BI in diabetic patients with PVD. Lack of FGF may inhibit the new vascularization process that takes place in the endothelial inflammation as shown in this study with the rise of the ICAM and VCAM the endothelial dysfunction biomarkers.

In the present study, pro- inflammatory molecules as ICAM-1 and VCAM-1 were markedly elevated in diabetic patients with PVD and had a negative correlation with A/BI in vasculopathy. This confirms their causative role in deficiency of peripheral blood flow. Hyperglycemia and glycosylation end products act as promoters for adhesion molecules expression in diabetic patients with and without asymptomatic peripheral artery occlusion [33].

Elhad et al. [33] stated that Adhesion molecules act as precursors of intimal and vascular smooth muscle cell proliferation and the elevated ICAM suggest a role for activated granulocytes in initiation of peripheral artery occlusion, depending of this evidence we can state that elevated ICAM and VCAM are inducers for capillary occlusion in diabetic vasculopathy shown in this study.

The finding that there was significant increase of plasma ICAM and VCAM in diabetic vasculopathy , the negative correlation between them and A/BI and that ICAM and VCAM could be predictors for peripheral blood flow in diabetic vasculopathy could be elucidated by the fact that ICAM has a role in atherosclerotic peripheral artery disease in diabetes, elevated levels of I-CAM and E-selectin in type 2 diabetes occur early in the course of asymptomatic peripheral artery occlusive disease and this is related to glycemic control [33], this fact could explain the rise of ICAM and VCAM levels in diabetic patients with vasculopathy and a bad glycemic control in this study. Previous studies stated that there is a rise of ICAM-1 levels especially in metabolically poorly controlled diabetic patients and those are at high risk of atherosclerosis and vascular complications [33].

The key contributing factors to vasculopathy in diabetes are oxidative stress, inflammation and cell adhesion, also the inflammatory protein C-reactive protein (CRP), and soluble intracellular adhesion molecule sICAM-1 and sE-selectin as indicators of endothelial activation. A combination of diet and exercise ameliorates oxidative stress, inflammation, and monocyte-endothelial interaction. Advanced glycation end products (AGEs) can also lead to an increase of vascular endothelial growth factor (VEGF) in retinal pigment epithelial cells, resulting in an increased ICAM-1 and VCAM-1 expression [34].

## Conclusions

According to the above results, for the first time the findings of the present study declare the direct association between the AM levels, b-FGF and AB/I which is a marker of arterial peripheral blood flow in diabetic PVD. Our study support the notion of the compensatory vasculo-protective role of AM which could have a direct impact on blood flow as it has an angiogenic and anti-inflammatory properties which act in relieving endothelial insults and stress. Down expression of b-FGF may be a predi sposing factor in microvascular derangement and low peripheral blood flow in PVD. The rise of ICAM-1 and VCAM-1 in vasculopathy and their negative correlation with A/BI support the role of inflammation in pathogenesis of diabetic PVD and indicates that inflammation could be the cause of reduced vascular integrity and contribute to vascular complications in diabetes. The results showed that diabetic vasculopathy is the result of multiple factors, so it is optimistic to believe that it could be targeted by drug interventions. The therapeutic use of AM and b-FGF as they have a direct impact on the peripheral blood flow, angiogenic and anti-inflammatory factors in the future may be prospective , they may be used to reduce the morbidity of cardiovascular and cerebrovascular attacks in diabetes through normalizing vascular endothelium function. b- FGF and endothelial dysfunction markers present in the systemic circulation appear to be suitable non-invasive predictors for assessing of peripheral blood flow in diabetic PVD.
